# Abnormal Airway Mucus Secretion Induced by Virus Infection

**DOI:** 10.3389/fimmu.2021.701443

**Published:** 2021-09-28

**Authors:** Yao Li, Xiao Xiao Tang

**Affiliations:** ^1^ State Key Laboratory of Respiratory Disease, National Clinical Research Center for Respiratory Disease, National Center for Respiratory Medicine, Guangzhou Institute of Respiratory Health, The First Affiliated Hospital of Guangzhou Medical University, Guangzhou, China; ^2^ Guangzhou Laboratory, Bio-island, Guangzhou, China

**Keywords:** virus, airway mucus secretion, pathogenesis, SARS-CoV-2, signaling pathway

## Abstract

The airway mucus barrier is a primary defensive layer at the airway surface. Mucins are the major structural components of airway mucus that protect the respiratory tract. Respiratory viruses invade human airways and often induce abnormal mucin overproduction and airway mucus secretion, leading to airway obstruction and disease. The mechanism underlying the virus-induced abnormal airway mucus secretion has not been fully studied so far. Understanding the mechanisms by which viruses induce airway mucus hypersecretion may open new avenues to treatment. In this article, we elaborate the clinical and experimental evidence that respiratory viruses cause abnormal airway mucus secretion, review the underlying mechanisms, and also discuss the current research advance as well as potential strategies to treat the abnormal airway mucus secretion caused by SARS-CoV-2.

## Introduction

In normal airways, the steady-state environment of mucus production, mucociliary clearance (MCC), and innate immune defenses play key roles in protecting respiratory system from inhaling environmental harmful substances including microbials (1). However, in the infected airways, excessively secreted pathological mucus may block MCC and air flow, resulting in airway obstruction and even respiratory distress (2). Mucins, the major component of mucus, assist eliminating inhaled pathogens under normal circumstances. Mucin hypersecretion and changes in mucin macromolecules form dysfunctional mucus gels often leading to airway obstruction and airflow limitation. Mucus hypersecretion, accumulation, and increased mucus viscosity largely influence disease development and prognosis. Respiratory viral infections are the leading causes of acute respiratory disease in all the age groups of healthy individuals. In view of the seasonal prevalence, population susceptibility, as well as relative comprehensiveness of the data, we have chosen several common and representative viruses, such as influenza viruses (IV) of the Orthomyxoviridae family, respiratory syncytial virus (RSV), parainfluenza virus (PIV) and human metapneumovirus (hMPV) of the Paramyxoviridae family, rhinovirus (RV) of the Picornaviridae family, severe acute respiratory syndrome coronavirus (SARS-CoV), and severe acute respiratory syndrome coronavirus 2 (SARS-CoV-2) of the Coronavirus family.

**Figure 1:**
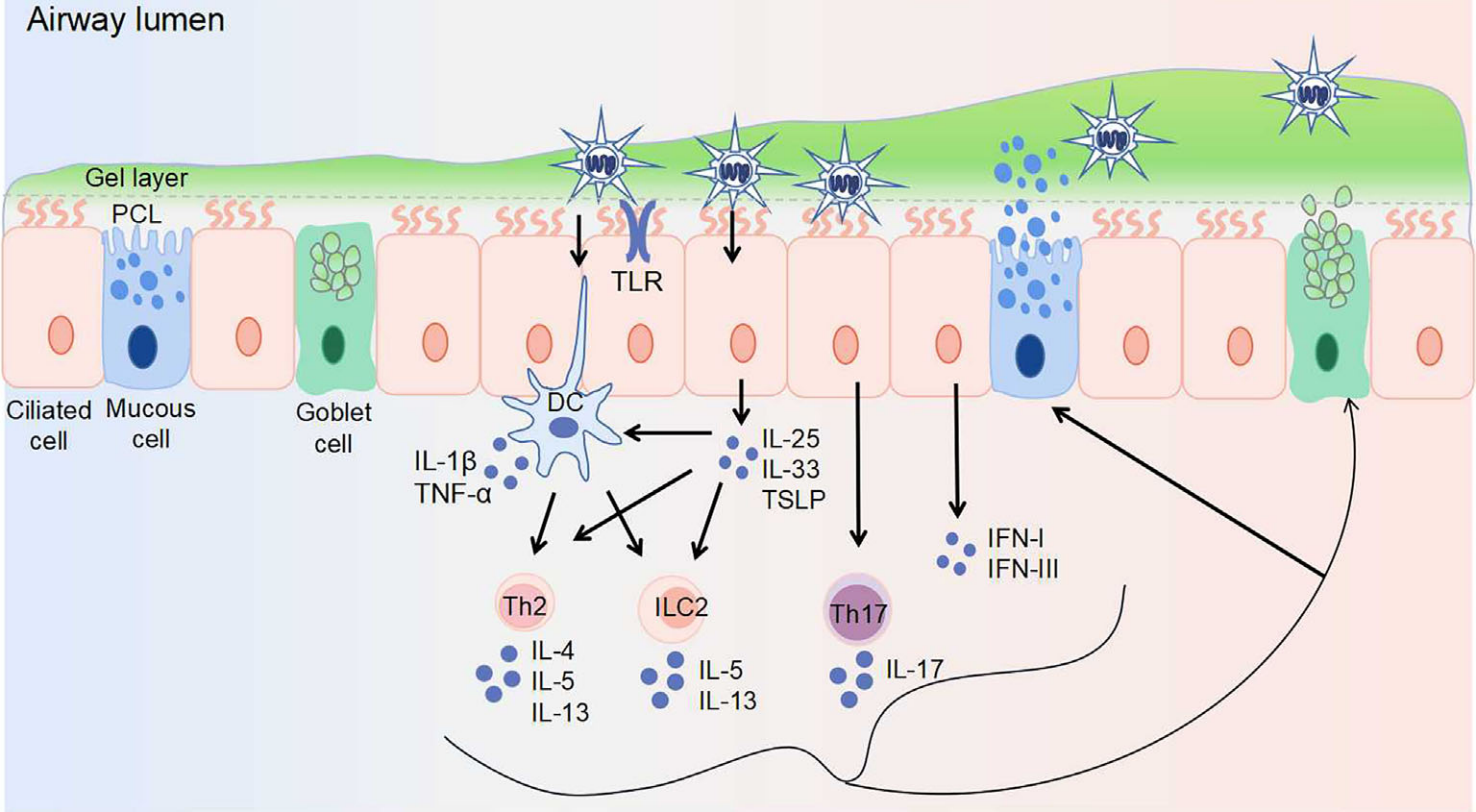
Virus invasion induces abnormal airway mucus secretion. The airway epithelium is the first line of defense, protecting the airway against pathogens. Airway surface liquid consists of two layers: the gel layer and periciliary liquid layer (PCL). The gel layer mainly contains secreted mucins MUC5AC and MUC5B, which are produced by goblet cells and mucous cells within submucosal glands (not shown), respectively. With Toll-like receptors (including TLR3, TLR4, TLR7) expressed on the surface, epithelial cells recognize viruses and elicit immune responses. The responses to virus-induced mucin overexpression: 1. epithelial cells secret IL-25, IL-33, TSLP, IFN-I, and IFN-III upon viral infection. The secretion of IL-25, IL-33, and TSLP activates Th2 cells, ILC2s, and dendritic cells. 2. dendritic cells activate Th2 cells and ILC2s, as well as secret IL-1β and TNF-α. 3. Th17 cells are stimulated to produce IL-17. Th2 cells are stimulated to produce IL-4, IL-5, and IL-13. ILC2s are stimulated to produce IL-5 and IL-13. These secreted cytokines further induce mucin exprssion and mucus secretion. DC, dendritic cell; IFN, interferon; IL, interleukin; ILC, innate lymphoid cell; PCL, periciliary liquid layer; Th, T helper cell; TLR, toll-like receptor; TNF-α, tumor necrosis factor-alpha; TSLP, thymic stromal lymphopoietin.

Virus invasion promptly stimulates innate defense response, with the activation of macrophages, epithelial cells, and dendritic cells. Different viruses may induce abnormal mucus secretion by activating different immune cells and cytokines (**Figure 1**). For instance, excessive accumulation of macrophages and neutrophils with an elevated expression of interleukin (IL)-1, IL-6, and tumor necrosis factor-alpha (TNF-α) is a typical feature of highly pathogenic IV infection (3, 4). It is reported that IL-1 and TNF-α serve vital roles in regulating mucin expression (5, 6). RSV, RV and hMPV infection in young children mainly activate T helper (Th) 2 immune responses, which stimulate type 2 innate lymphoid cells (ILC2s) and Th2 cells to secrete Th2 cytokines including IL-4, IL-5, and IL-13 (7). In contrast, during severe SARS-COV-2 infection, unphysiologically reduced levels of ILCs in the circulation were observed (8). The first-line signals including interferon (IFN) signals and NOD-like receptor family pyrin domain containing 3 (NLRP3) inflammasome may be the most dominant, explained by the uncoordinated IFN responses that amplify TNF/IL-1β-centered hyperinflammatory signatures in severe COVID-19 patients (9). Similar to SARS-COV-2, dysregulated type I IFN response combined with inflammatory monocyte–macrophage responses are considered as a crucial cause of severe SARS-CoV infection (10, 11). Thus, IFN response plays a vital role during SARS-CoV and SARS-CoV-2 infections. In addition, IL-17 produced by Th17 cells is thought to be associated with mucus hypersecretion during RSV and SARS-COV-2 infections.

The pathogenesis underlying virus infection induced abnormal airway mucus secretion is far from satisfactory. Here, we review the clinical and experimental evidence of virus-induced abnormal airway mucus secretion as well as summarize the current understanding of the underlying mechanisms (**Tables 1**, **2**), which may suggest new therapeutic strategies.

**Table 1:** Clinical features of virus-induced abnormal airway mucus secretion.

Virus	Sample	Changes in mucus quantity/properties	Reference
IV	Patients	Productive cough, increased nasal discharge, increased sputum	(12)
	Patients' autopsies	Trachea and bronchus mucosal surface: swelling and mucous gel material	(13)
	H7N9 patients (median age: 54 years)	Yellow sputum	(14)
	H1N1 patients (median age: 48 years)		
	IAV patients' sputum (age <20 years)	Increased mucus viscosity	(15)
RSV	Patients	Increased mucus secretion in both quantity and viscosity, airway lumen: mucus mixed with cellular debris and fibrin	(16)
	Hospitalized adults (median age: 80 years)	Sputum production	(17)
	Children's autopsies	Enhanced mucus secretion, bronchiolar luminadense: thick plugs composed of cell debris, strands of fibrin, mucus, and a material with the DNA staining properties	(18)
	Well-differentiated primary pediatric bronchial epithelial cells (WD-PBECs)	Increased mucus secretion, goblet cell proliferation, broken cilia cells, increased MUC5AC expression	(19, 20)
RV	Patients	Bronchioles: increased mucus secretion, cell debris	(21)
	Sputum (median age of patients: 24 years)	Sputum: total cells, total leukocytes and neutrophils increase	(22)
HPIV	Hospitalized adults (median age: 60.4 years)	Sputum production	(23)
	HPIV4 children (median age: 2.3 years)	Rhinorrhea, cough followed by sputum	(24)
	HPIV1 patients	Nasal discharge, even become mucopurulent	(25)
HMPV	Children (age <18 years)	Sputum production, rhinorrhea	(26, 27)
	Children's BAL and lung Biopsy samples (age: 1 to 16 years)	BAL: mucus, epithelial cell degeneration or necrosis with detached ciliary tufts and round red cytoplasmic inclusions	(28)
	Cynomolgus macaques	Suppurative rhinitis, bronchial lumen: a few sloughed ciliated epithelial cells, cell debris and mucus	(29)
SARS-CoV	Patients' autopsies (median age: 46 years)	Bronchus: mucopurulent material	(30)
SARS-CoV-2	Critical ill patients' sputum	Bronchioles: mucus retention, respiratory tract: high-viscosity white to gray sputum	(31)
	85-year-old man's autopsy	Alveoli: gray-white highly viscous exudates	(32)

**Table 2:** Mechanisms of virus-induced abnormal airway mucus secretion.

Virus	Animal species/cell line	Evaluation method	Mechanism	Reference
IV	A549 cells	RT-PCR, flow cytometry, western blot	IV→NF-κB, MAPK p38→MUC5AC↑, IV→ TNF-α↑	(33)
	BALB/c mice	RT-PCR, qPCR, ELISA, flow cytometry	H1N1→TLRs-TNF-α-NF-κB→mucus hypersecretion	(34)
	NCI-H292 cells	RT-PCR, ELISA, immunohistochemistry	IAV→EGFR-MEK/ERK-Sp1→MUC5AC↑	(35)
RSV	B6 wildtype mice	qPCR, flow cytometry	RSV→TLR7→IL-4, IL-13, IL-17→MUC5AC, gob5↑	(36)
	BALB/c mice	RT-PCR, ELISA, flow cytometry	RSV→IL-17→MUC5AC, gob5↑	(37)
	BALB / cJ mice	RT-PCR, western blot, histopathology, flow cytometry	RSV→IL-13, TNF-α→mucin↑	(38)
	DBA2/J mice	ELISA, PAS staining	RSV→IL-13→STAT6→mucus production↑	(39)
	C57B/6 mice	qRT-PCR, ELISA, PAS staining	RSV→TLR3→IL-13→gob5↑, mucus production↑	(40)
	Severe pneumonia children	RT-PCR, ELISA, western blot	RSV→JNK-AP-1→MUC5AC, MUC5B↑	(41)
	HBE cells	qRT-PCR, western blot, immunohistochemistry	RSV→AP-1→c-Jun→MUC5AC↑	(42)
	A549 cells	RT-PCR, ELISA	RSV→MAPK p38/JNK-AP-1-c-Jun/c-Fos→NF-κB→MUC1, MUC2, MUC5AC, MUC5B, MUC8↑	(43)
	BALB/c mice	ELISA, western blot, histopathology		(44)
	BALB/c mice	RT-PCR, immunofluorescence	RSV→TRPV1-PKC-NF-κB→IL-1β, IL-4, IL-5, IL-13, MUC5AC↑	(45)
RV	BALB/c mice	qPCR, ELISA, immunofluorescence, flow cytometry	RV→IL-25→ILC2s→IL-13→MUC5AC, MUC5B, gob5↑	(46)
	BALB/c mice	RT-PCR, flow cytometry, immunohistochemistry	RV→ IL-13→ MUC5AC, MUC5B, gob5↑	(47)
	BALB/c mice	RT-PCR, ELISA, immunofluorescence, flow cytometry	RV→IL-25, IL-33, TSLP→ILC2s→IL-13→MUC5AC↑	(48)
	C57BL/6J mice, NLRP3−/−mice	qPCR, western blot, flow cytometry, immunofluorescence	RV→NLRP3-caspase-1→IL-1β→IL-5, IL-13, IL-25, IL-33, MUC5AC, gob5↑	(49)
	HNECs	qPCR, western blot, immunofluorescence	RV→DDX33/DDX58-NLRP3-caspase-1→IL-1β, MUC5AC↑	(50)
	NCI-H292 cells	RT-PCR, western blot	RV→TLR3→TGF-α→EGFR-ERK→MUC5AC↑	(51)
	NCI-H292 cells	qPCR, ELISA, western blot	RV→TGF-α→EGFR-MEK/ERK-Sp1→MUC5AC↑	(52)
HMPV	A549 cells, BALB/cJ mice	qRT-PCR, histopathology	HMPV→IL-33, TSLP→ILC2s→IL-5, IL-13 and TNF-α↑	(53)
	BALB/c mice	cytometric bead array analysis, flow cytometry immunohistochemistry	HMPV→IL-6, TNF-α↑	(54)
SARS-CoV	C57BL/6J mice	RT-PCR, ELISA, flow cytometry, immunohistochemistry	SARS-CoV→TLRs→IL-6, TNF, IFN-γ↑	(55)
	EGFR(DSK5) mice	RT-PCR, western blot, immunohistochemistry	SARS-CoV→EGFR↑	(56)
SARS-CoV-2	HACE2-transgenic mice, PAECs/BEAS-2 B cells Patients	RT-PCR, ELISA, western blot, immunohistochemistry	SARS-CoV-2→IFN-β, IFN-γ→IDO-Kyn→AhR↑→mucins expression (MUC1, MUC4, MUC5AC, MUC5B, MUC16)↑	(57)
		ScRNA-seq	SARS-CoV-2→NF-κB, IL-1, TNF, AP-1→MUC5AC, MUC5B, MUC4, MUC16, MUC20↑	(58)
		Cytokine detection	SARS-CoV-2→IL-4, IL-6, IL-13, IL-17, TNF-α, IL-1β↑	(59)

→ Activation; ↑ Increase.

## Airway Mucus

Mucus, viscous fluid containing multiple components with complex selective permeability, is the first barrier of host defense. It is well recognized that the mucus layer consists of two layers: the gel layer and the periciliary liquid layer (PCL), one facilitates blocking the penetration of invading pathogens to the airway epithelium, while the other contributes to ciliary beating and MCC (60). The surface epithelium close to the mucus layer is composed of two major cell types: ciliated cells and secretory cells (goblet cells, mucous cells) (**Figure 1**). Goblet cells have secretory granules that store and secrete mucins, while ciliated cells are responsible for the transportation of secreted mucus (61).

There are 16 mucins in the human respiratory tract (62). The gel layer mainly contains secreted mucins MUC5AC and MUC5B, which are produced by goblet cells and mucous cells within submucosal glands, respectively. The polymer network formed with these mucins can permit/limit the passage of molecules and nanoparticles (63). The PCL mainly contains membrane-tethered mucins MUC1, MUC4, MUC16, and MUC20. In addition to these mucins, respiratory epithelial cells secrete a large number of non-mucin proteins, including defensins, enzymes, antibacterial peptides, immunoglobulins, protease inhibitors, and oxidants (64). Altogether, these components constitute the complex physiological structure of the mucus layer. Virus invasion breaks the homeostasis of the mucus layer, contributing to airway obstruction, remodeling, and disease progression. Mucus may, in turn, affect the penetration, mobility, and transmissibility of viruses. Moreover, the mucus–virus interaction may also be dependent on the type of virus (60, 65).

The importance of mucin in the mucus layer should not be ignored and mucin abnormalities may contribute to lung disease (66, 67). MUC5B^-/-^ mice showed abnormal lung defense and reduced MCC, leading to impaired upper airway obstruction (68). This indicated that MUC5B is required for MCC and controlling infections in the airways, whereas MUC5AC is dispensable. A study in IV-infected MUC5AC transgenic mice uncovered the protective effect of MUC5AC (69). Interestingly, contrary to the protective roles of MUC5AC mentioned above, another study showed its harmful role in enhancing pulmonary inflammation during acute lung injury (70). The proportion of MUC5AC and MUC5B varies in different states of health in humans. The results from asthmatic sputum samples indicate an increase in the relative amount of MUC5B 5 times over MUC5AC (71). Similar findings during allergic mucous metaplasia in mice showed an increased production of both MUC5AC and MUC5B, while MUC5AC mRNA level is present at 30-fold lower than MUC5B (72). One study revealed distinct morphology and interactions for MUC5B and MUC5AC, suggesting that the two mucins make distinct contributions to mucociliary transport (73). Therefore, differing levels of MUC5B or MUC5AC may impact specific outcome on disease, and this needs further in-depth investigation in different disease models.

## Influenza Virus

Influenza virus (IV), belonging to the Orthomyxoviridae family, is responsible for the flu season each year and can cause severe respiratory infections. Clinical studies showed that IV infections cause respiratory symptoms such as productive cough, increased nasal discharge, and sputum (12, 74). According to a series of autopsies performed in 1918, the trachea and bronchi of the patients are invaded, and the mucosal surface is obviously red and swollen, sometimes covered with mucous gel material, and there are also edema and hyperemia of the submucosa (13). Both H7N9 and H1N1 patients appeared yellow sputum symptoms (14). In addition, airway mucus viscosity of influenza A virus-infected patients is significantly higher than that of normal saline (15). The changes in mucus properties are related to the increased mucin expression during IV infection (68, 69, 75).

The family of Toll-like receptors (TLRs), expressed on dendritic cells and macrophages, serves as key pattern recognition receptors (PRRs) of innate immune system and promotes production of inflammatory cytokines and chemokines as well as expression of mucin genes. Shi et al. found that the H1N1 IV stimulates lung tissue swelling and excessive mucus production *via* TNF-α stimulated nuclear factor kappa B (NF-κB) activated by TLRs. In addition, TNF-α inhibitor etanercept was found to reduce lung damage and excessive mucus secretion in IV-infected mice (34). Mata et al. found that influenza viruses (influenza virus A and B) induce MUC5AC expression in A549 cells through NF-κB nuclear translocation and mitogen-activated protein kinase (MAPK) p38 phosphorylation (33). Besides, Barbier et al. showed that MUC5AC is stimulated through epidermal growth factor receptor (EGFR)-mitogen-activated protein kinase (MEK)/extracellular regulatory kinase (ERK)-specific protein 1 pathways in IV-infected NCI-H292 cells (35). These results indicated involvement of multiple signal pathways in IV-induced airway mucus alterations.

## Respiratory Syncytial Virus

Respiratory syncytial virus (RSV), belonging to the Paramyxoviridae family, is the main cause of bronchiolitis and viral pneumonia, as well as worsening asthma in children. The main susceptible population of RSV is infants (less than 6 months). Compared with adults, the innate immune system of infants is immature, explained by a partial defection of mucosal innate responses (lack of TLRs, type I and type III interferons). These molecules are “sentinels” of the innate immune system, which induce production of cytokines and chemokines, thereby activating and attracting innate lymphocytes, granulocytes, dendritic cells, and monocytes to the site of infection (16).

During RSV infection, the quantity and viscosity of mucus both increase, and severe obstruction of the airway lumen occurs mixed with cellular debris and fibrin (16). Lee et al. found that 81.2% of the RSV-infected patients had sputum production (17). In histopathological studies of RSV-infected children, mucus secretion is enhanced, with thick plugs composed of cell debris, strands of fibrin, and a material with the DNA staining properties in the bronchiolar lumina (18). Besides, studies showed that RSV infection destroyed cilia cells induced mucus hypersecretion and goblet cell hyperplasia, as well as stimulated MUC5AC expression (19, 20).

Studies showed that the increased production of IL-17, as well as Th2 cytokines IL-4 and IL-13 correlated with the increased mucus gene expression and goblet cell hyperplasia during RSV infection (36, 37). IL-17 was reported to stimulate the expression of MUC5AC and MUC5B *via* activating the JAK2–STAT3–ERK signaling pathway (76). Besides, the elevated MUC5AC level during RSV infection is associated with TNF-α and the IL-13–STAT6 pathway (38, 39). IL-13, a potent inducer of excessive mucus production and mucus metaplasia, stimulates MUC5AC expression through JAK–STAT6–ERK or JAK–STAT6–IKK–NF-κB pathways (77). In the studies of animal infection models, RSV-infected TLR3^-/-^ mice demonstrated significant elevated mucus production in the airways, which is associated with an increased expression of gob5 (a putative calcium-activated chloride channel thought to regulate mucus production and/or secretion), while depletion of IL-13 reduces gob5 expression in TLR3^-/-^ mice (40). Similarly, IL-17 neutralization of RSV-infected TLR7^-/-^ mice had a clear decrease in mucus hypersecretion, as well as the expression of MUC5AC and gob5 (36). Taken together, Th2 and Th17 immune responses activated by TLRs may be the leading cause of RSV-induced mucus hypersecretion.

Besides, RSV-induced mucus overproduction was thought to be related to NF-κB pathways, which coordinate activation of various signaling pathways that regulate proinflammatory cytokines expression (such as IL-1, TNF-α) (78). Several studies showed that the elevated expression of MUC5AC and MUC5B during RSV infection is associated with the c-Jun N-terminal kinase (JNK)–activator protein-1 (AP-1)–c-Jun signaling pathway (41, 42). Lee et al. found that RSV induces NF-κB through the MAPK p38/JNK-AP-1 signaling pathway, thereby inducing the expression of various mucins, including MUC1, MUC2, MUC5AC, MUC5B, and MUC8 (43). Besides, Jing et al. found that RSV induces MUC5AC expression and excessive airway mucus secretion in mice *via* the transient receptor potential vanilloid 1 (TRPV1)–protein kinase C (PKC)–NF-κB pathway (44). Another study demonstrated that inhibiting the TRPV1–PKC–NF-κB pathway reduces excessive mucus secretion (79). Together, NF-κB seems to be a pivot associated with regulation of MAPK and TRPV1 pathways during RSV-induced mucus hypersecretion.

Other than TLRs and NF-κB pathways, RSV-induced MUC5AC expression is also associated with the EGFR pathway, and inhibition of EGFR suppresses RSV-induced airway mucin expression in mice (45). As the EGFR pathway plays an important role in IV-induced mucin overexpression as well, we speculate that it may be a common pathway underlying virus-induced mucus hypersecretion.

## Rhinovirus

Human RV, belonging to the Picornaviridae family, is the major cause of common cold. People of all ages are generally susceptible to RV. Increased mucus secretion and cell debris are found in the bronchioles of RV-infected infants, leading to mechanical airway obstruction (21). In the lower respiratory tract, patients with RV infection showed remarkable elevation of total cells, leukocytes, and neutrophils in sputum (22).

During RV infection, the level of mucus-related genes (such as MUC5AC, MUC5B, and gob5) is elevated, leading to excessive mucus secretion (46–48). Given the similarities in pathogenicity between RSV and RV, some common mechanisms of the virus-induced mucus hypersecretion can be found. Zhu et al. showed that RV activates the EGFR–ERK signaling pathway *via* mediating TLR3, thereby inducing MUC5AC expression, and the TLR3-mediated pathway partially depends on TRIF and is negatively regulated by myeloid differentiation primary response protein 88 (51). A similar study found that RV activates EGFR–MEK/ERK–Sp-1 signaling cascade to promote MUC5AC expression (52). Both studies showed that RV-induced MUC5AC requires TGF-α release upstream of EGFR activation. Together, it is clear that the EGFR pathway plays a crucial role in the mucus hypersecretion induced by IV, RSV, and RV.

Airway epithelial cells are sources of the cytokines IL-25, IL-33, and thymic stromal lymphopoietin (TSLP), which impact the expansion of ILC2s to enhance Th2 inflammation. Studies found that IL-25, IL-33, and TSLP cooperate in the expansion of ILC2s and mucous metaplasia in RV-infected immature mice, and IL-13 produced by ILC2 is associated with MUC5AC expression (46–48). It appears that RSV and RV infections in young children stimulate inflammation with Th2 cell characteristics, leading to abnormal mucus production.

NLRP3 inflammasomes are another class of PRR in the innate immune recognition system. Liu et al. found that RV infection upregulates IL-1β secretion and MUC5AC expression in human nasal epithelial cells through DDX33/DDX58–NLRP3–caspase-1 pathway, thereby inducing mucus production. Furthermore, treatment with IL-1β significantly enhances MUC5AC expression in these cells (50). Also, Han et al. found that RV infection activates lung inflammasome expression in mature mice, including NLRP3, IL‐1β, and caspase-1 (49). In addition, as compared to RV-infected wild-type mice, the expressions of IL-5, IL-13, IL-25, IL-33, MUC5AC, and gob5 in RV-infected NLRP3^-/-^ and IL‐1β^-/-^ mice were significantly increased. These results demonstrated that inhibition of NLRP3 and IL‐1β increases RV-induced type 2 immune responses and mucin expression. Also, TNF-α and IL-1β were reported to induce MUC5AC overexpression *via* MAPK p38 activation (80). All these suggested that the NLRP3 inflammasome pathway and IL-1β play vital roles in cellular alterations and mucin overproduction during RV infection.

## Parainfluenza Virus

PIV, a single-stranded RNA virus belonging to the paramyxovirus family, is the cause of seasonal lower respiratory infections in infants, children, and immunocompromised people. The most common clinical presentations of PIV infection are cough, sputum production, and rhinorrhea (23, 24, 81). In addition, there was a typical nasal discharge that often became mucopurulent and lasted for 1–2 weeks (25). The data are limited and clarification of the mechanism of PIV-induced mucus hypersecretion requires more investigation.

## Human Metapneumovirus

hMPV, discovered in 2001, belonging to the paramyxovirus family, is similar to RSV in structure and pathogenicity. Children infected with hMPV most commonly exhibited upper respiratory symptoms such as cough, rhinorrhea, and sputum (26, 27). Bronchoalveolar lavage fluid (BAL) samples from hMPV-infected children showed epithelial cell degeneration or necrosis with detached ciliary tufts and round red cytoplasmic inclusions, neutrophils, and mucus (28). Pathological examination in hMPV-infected cynomolgus macaques demonstrated suppurative rhinitis, and a few sloughed ciliated epithelial cells, cell debris, and mucus in bronchial lumen (29).

HMPV infection significantly increased expression of MUC1, MUC2, MUC4, MUC5AC, and MUC19 in both normal human bronchial epithelial cells and BALB/c mice (82). A recent study compared the differences in mucin expression between hMPV and RSV infections. HMPV induced a stronger response of MUC2, MUC5AC, and MUC5B while RSV induced a 200-fold increase of MUC8 as compared to hMPV infection (83). Though the data on the mechanisms of hMPV-induced mucus hypersecretion are limited, the similarities in clinical presentation between hMPV-infected infants and RSV-infected infants may suggest some potential common mechanisms. Similar to RSV, hMPV activates TSLP and IL-33 to induce Th2 and Th1 responses (IL-5, IL-13 and TNF-α) (53). In addition, hMPV induced higher levels of IL-6 and TNF-α as compared to RSV infection (54). Thus, we speculate that hMPV-induced mucus hyersecretion is partly related to Th2 and Th1 responses.

## Severe Acute Respiratory Syndrome Coronavirus

Severe acute respiratory syndrome (SARS) is an acute lung infection caused by SARS-CoV. The population is generally susceptible to SARS-CoV, mostly adults between 25 and 50 years old, but the mortality rate of patients aged 60 years or older is as high as 43% (84). Coronavirus can trigger severe alveolar damage and the most typical pathological finding in the advanced stage of SARS is diffuse alveolar damage, accompanied by extensive fibrin exudation, edema, and inflammatory infiltration (30). At the same time, mucopurulent material was detected in the upper respiratory tract bronchus (10).

As mentioned above, the TLR-activated EGFR pathway is closely correlated with mucus hypersecretion induced by IV, RSV, and RV. A study found that SARS-CoV infection upregulates the EGFR pathway (56). Another study showed that EGFR activation induces mucus hypersecretion *via* promoting MUC5AC expression (85). Therefore, we speculate that SARS-CoV may induce airway mucus hypersecretion *via* the EGFR pathway, sharing a common mechanism with IV, RSV, and RV. Besides, it was demonstrated that the downstream of TLR3 signaling, such as IL-6, TNF, and IFN-γ, are increased during SARS-CoV infection (55). Whether these cytokines also play a role in SARS-CoV-induced airway mucus hypersecretion remains uncertain and awaits further investigation.

## Severe Acute Respiratory Syndrome Coronavirus 2

Similar to SARS-CoV, individuals of all age groups are susceptible to SARS-CoV-2. Epidemiological analysis showed that 87% patients with COVID-19 are 30 to 79 years of age, and the elderly (>60 years old) are more likely to develop severe respiratory disease while young adults and children only have mild symptoms (86, 87). Mucus-like substances were found in the BAL from COVID-19 patients (57). Also, mucus retention was observed in the bronchioles of COVID-19 patients, and white to gray sputum with high viscosity was aspirated from the respiratory tract (31). The high-viscosity sputum was considered to be related to the elevated MUC5AC expression. According to the autopsy report of a COVID-19 patient, plenty of gray-white highly viscous exudates were seen in the lungs overflowing from the alveoli (32).

The levels of MUC5AC and MUC5B as well as membrane mucins MUC4, MUC16 and MUC20 are elevated in the lung club cells of COVID-19 patients (58). IFN signals are the first-line signals that are responsible for initiating the rapid antiviral response as well as presenting the antigen to adaptive immune cells upon viral invasion. Plasmacytoid dendritic cells are the main source of type-I IFN. Liu et al. found that IFN-β and IFN-γ upon SARS-CoV-2 infection activate the aryl hydrocarbon receptor (AhR) through the indoleamine-pyrrole2,3-dioxygenas (IDO)–Kynurenine pathway, thereby inducing the expression of MUC1, MUC4, MUC5AC, MUC5B, and MUC16 (57). Giovannoni et al. have confirmed AhR as a candidate therapeutic target for viral infection. AhR antagonists improve lung pathology by enhancing antiviral immunity, as well as inhibiting excessive mucus production (88). Therefore, blocking IFN signaling and AhR activity may be a potential strategy to reduce virus-induced airway mucus production and accumulation.

Besides, SARS-CoV-2 increases the expression of proinflammatory cytokines, such as IL-4, IL-6, IL-13, IL-17, TNF-α, and IL-1β (59). Single-cell RNA sequencing revealed that SARS-CoV-2 infection stimulates IL-1 and TNF response, as well as NF-κB and AP-1 enrichment (58). These data indicated that SARS-CoV-2 induces mucin secretion potentially *via* IL-1β and TNF-α. It is reported that pro-inflammatory factor IL-1β upregulates MUC5AC expression (5). Busse et al. found that long-term exposure to TNF-α increases the expression of MUC5AC and gob5 in mice (6). These studies reveal a potential role of IL-1β and TNF-α inhibitors in treating SARS-CoV-2-induced excessive mucus production and secretion. Feldmann et al. suggested initiating anti-TNF therapy in COVID-19 patients as early as possible (89). In addition, Kyriazopoulou et al. found that IL-1 blockade (anakinra) not only blocks IL-1α and IL-1β but also decreases IL-6 production, indicating that it may be a potential treatment for patients with COVID-19 (90). Besides, a recent study has shown that the anti-inflammatory drug melatonin inhibits NF-κB cascades and NLRP3 inflammasome, the two main pathways during SARS-CoV-2 infection. What is more, phase II clinical trials of melatonin in COVID-19 patients are currently underway (91, 92).

Recent studies have confirmed the important role of IL-17 in the pathogenesis of COVID‐19 disease (93). JAK2 inhibitor Fedratinib has been proved to inhibit the production of several Th17 cytokines, including IL-6, IL-17, IL-21, IL-22, IL-1β, and TNF-α, thereby preventing Th17-related cytokine storm in COVID-19 patients (94). Therefore, modulating IL-4/IL-13/IL-17 signaling pathways may be a therapeutic strategy for SARS-CoV-2-induced mucus hypersecretion. Also, Zhang et al. found that traditional Chinese medicine expectorants regulate MUC5AC expression *via* IL-17 and TNF signaling pathways, which are related to virus infection such as influenza virus A and human cytomegalovirus infections (95). They speculated that core herbs of the phlegm-eliminating recipe is a potential therapy in COVID-19-induced mucus hypersecretion as well. Further experiments are needed to clarify the mechanisms of these herbs.

## Conclusions and Future Perspective

**Figure 2:**
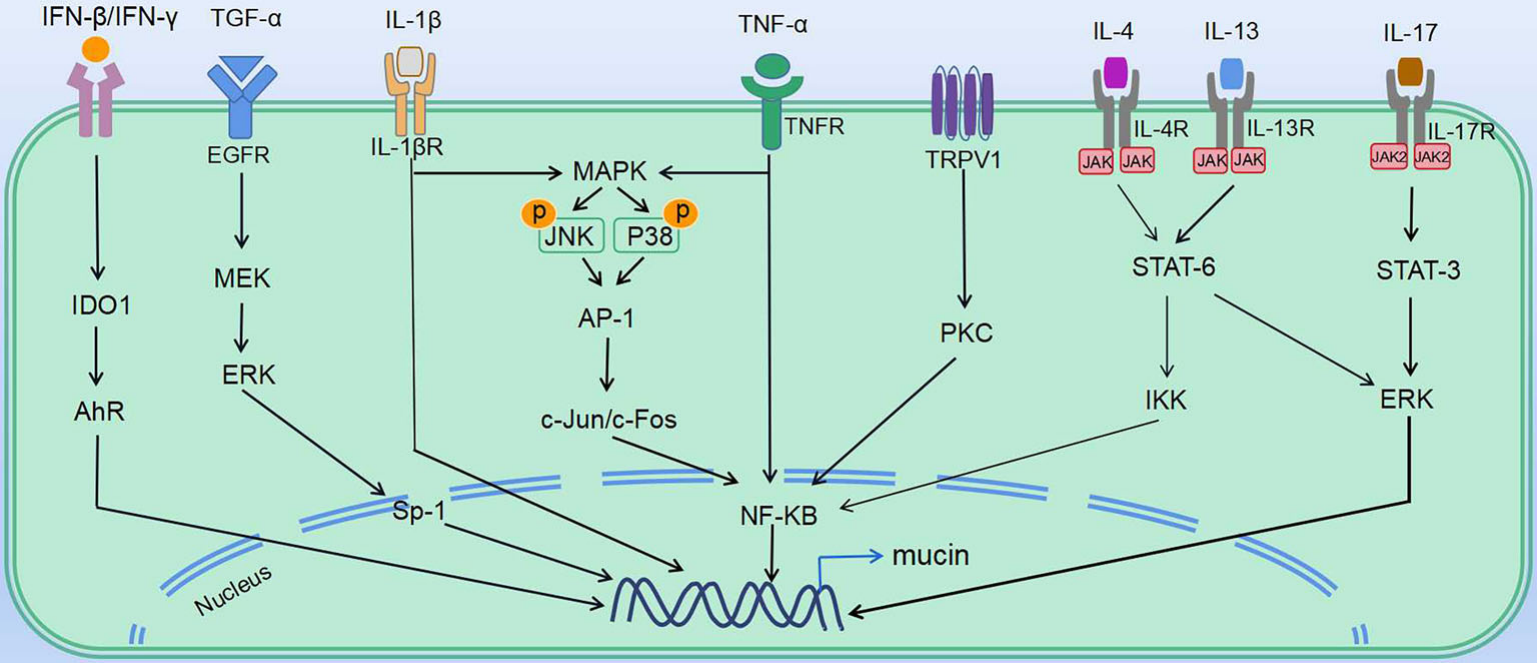
Mechanisms underlying virus-induced abnormal mucus secretion. Regulation of virus-induced abnormal mucus secretion: various mediators affect the transcriptional regulation of mucin gene expression in mucin secretory cells (including goblet cells and mucous cells). Multiple ligands (TGF-α, IFNs, TNFα and ILs) induce downstream signaling, which leads to expression of mucins. IV induces mucin expression *via* TNF-α, as well as actives EGFR and MAPK pathways. RSV induces mucin expression through activating expression of IL-1β, IL-4, IL-5, IL-13, IL-17 and TNF-α, as well as MAPK pathways. RV induces IL-13 expression, as well as actives EGFR and TRPV1 pathways. HMPV induces expression of IL-13 and TNF-α. SARS-CoV induces TNF-α expression, as well as actives EGFR pathway. SARS-CoV-2 induces mucin expression *via* IFN-β, IFN-γ, IL-4, IL-13, IL-17, TNF-α, and IL-1β.

In vivo and *in vitro* studies have shown that respiratory viruses induce abnormal airway mucus secretion through common as well as distinctive mechanisms (**Tables 2**, **3** and **Figure 2**). Regulation of these signaling cascades is critical for the treatment of virus-induced abnormal airway mucus secretion.

**Table 3:** Signal pathways of virus-induced abnormal airway mucus secretion.

Signal pathway	IV	RSV	RV	HMPV	SARS-CoV	SARS-CoV-2
NLRP3-caspase-1→IL-1β-CREB, NF-κB			√			√
MAPK p38/JNK-AP-1-c-Jun/c-Fos-NF-κB	√	√				
EGFR-MEK/ERK-Sp1	√	√	√		√	
TNF-α-NF-κB	√	√		√	√	√
IL-4/IL-13→JAK-STAT6-ERK/IKK-NF-κB		√	√	√		√
IL-17→JAK2-STAT3-ERK		√	√			√
TRPV1-PKC-NF-κB		√				
IFN-IDO-Kyn-AhR						√

PRRs are involved in activating innate immune signaling, including NLRP3 inflammasome and TLR pathways, which rapidly recognize viral components and induce production of cytokines and antiviral molecules. As observed in IV, RSV, and SARS-CoV, the TLR pathway is activated and contributes to mucus hypersecretion. RV and SARS-CoV-2 were both reported to induce NLRP3 inflammasome. Besides, cytokines share some common mechanisms. Th1, Th2, and Th17 cytokines such as IL-5, IL-13, and IL-17 are induced *via* JAK–STAT pathways. Th2 and Th17 immune responses are thought to be dominant during RSV and RV-induced mucus hypersecretion. Also, the importance of NF-κB cascades should not be ignored. NF-κB, the downstream of various signaling pathways during viral infection, can be activated by the TLR pathway, TNF-α, JAK–STAT pathways, as well as MAPK signaling. Moreover, EGFR, the vital node of the pathway, controls several downstream cascades and serves as a common mechanism in stimulating abnormal mucus secretion during IV, RV, RSV, and SARS-CoV infection (**Table 3**). Intervening the signaling molecules in the EGFR cascade could block exaggerated mucin expression.

Other than the common mechanisms, some viruses induce mucus secretion *via* distinctive pathways. For instance, IFN-β or IFN-γ induced by SARS-CoV-2 upregulates mucin expression through the indoleamine-pyrrole2,3-IDO–Kynurenine–AhR pathway, while RSV-induced mucus hypersecretion is associated with the TRPV1-regulation of NF-κB and MUC5AC. As the data on the distinct mechanisms between pathogens are rather limited, further study is needed to define why these viruses induce abnormal airway mucus secretion through unique mechanisms. We speculate that the differences in immune response due to age/demographic as well as different types and features of the virus may at least partly explain the distinct mechanisms between pathogens.

Looking for strategies to treat abnormal mucus secretion in COVID-19 patients is crucial for disease development and prognosis. Although the mechanisms associated with SARS-CoV-2-stimulated mucus hypersecretion have not been fully elucidated, similarities can be found. The cues we summarized in **Figure 2** could be of help in developing therapeutic strategies for SARS-CoV-2-induced airway mucus hypersecretion.

A number of immune-related signaling pathways (NF-κB cascades, JAK–STAT pathway, IFNs pathway, and inflammasome signal pathway) are activated during virus-induced airway mucus hypersecretion and recent studies have highlighted some key involved molecules that have clinical relevance. Type-I IFN has been proved as an effective drug against SARS-CoV and is also considered as a potentially effective drug during early-life SARS-CoV-2 infection (4). What is more, the safety of IFN-I treatment has been assessed in independent clinical trials. Baricitinib, inhibiting multiple cytokine-signaling pathways through JAK–STAT, is considered as a potential strategy against SARS-CoV-2 infection (96, 97). Also, JAK2 inhibitor Fedratinib has been proved to decrease IL-17 expression in murine models, and clinical trials are needed to further verify its safety. Anakinra, an interleukin-1 receptor antagonist that blocks activity of IL-1α and IL-1β, is suggested as a therapeutic approach in COVID-19 patients due to its safety and efficiency. Melatonin, inhibitor of NF-κB cascades and NLRP3 inflammasome, has entered Phase II clinical trials for COVID-19 patients. Moreover, the antioxidant and anti-inflammatory drug N-acetyl-l-cysteine is considered as a candidate drug for virus-induced airway mucus hypersecretion (98–100). TNF blockers are well-evaluated drugs and have been used in autoimmune inflammatory diseases for more than 20 years. Thus, it is suggested that initial assessments of TNF blockers should be done in COVID-19 patients as soon as possible (89). AhR antagonists are still to be explored.

## Author Contributions

XT conceived and designed the manuscript, provided guidance, and edited the manuscript. All authors contributed to the article and approved the submitted version.

## Funding

This work was supported by the National High-Level Talents Program (XT), the National Natural Science Foundation of China (81770015, XT), the Local Innovative and Research Teams Project of Guangdong Pearl River Talents Program (2017BT01S155), the Open Project of State Key Laboratory of Respiratory Disease (SKLRD-OP-202109), the Special Fund for Science and Technology Innovation of Guangdong Province (2020B1111330001), and the Guangzhou Institute of Respiratory Health Open Project (funds provided by China Evergrande Group)-Project No. 2020GIRHHMS16.

## Conflict of Interest

The authors declare that the research was conducted in the absence of any commercial or financial relationships that could be construed as a potential conflict of interest.

## Publisher’s Note

All claims expressed in this article are solely those of the authors and do not necessarily represent those of their affiliated organizations, or those of the publisher, the editors and the reviewers. Any product that may be evaluated in this article, or claim that may be made by its manufacturer, is not guaranteed or endorsed by the publisher.

## Abbreviations

AhR, aryl hydrocarbon receptor; AP-1, activator protein-1; BAL, bronchoalveolar lavage fluid; COVID-19, coronavirus disease-19; EGFR, epidermal growth factor receptor; ERK, extracellular regulatory kinase; hMPV, human metapneumovirus; IDO, indoleamine-pyrrole2,3-dioxygenase; IFN, interferon; IL, interleukin; ILC2s, type 2 innate lymphoid cells; IV, Influenza virus; JNK, c-Jun N-terminal kinase; MAPK, mitogen-activated protein kinase; MCC, mucociliary clearance; MEK, mitogen-activated protein kinase; NF-κB, nuclear factor-kappa B; NLRP3, NOD-like receptor family and pyrin domain containing 3; PCL, periciliary liquid layer; PIV, parainfluenza virus; PKC, protein kinase C; PRRs, pattern recognition receptors; RSV, respiratory syncytial virus; RV, Rhinovirus; SARS, severe acute respiratory syndrome; SARS-CoV, severe acute respiratory syndrome coronavirus; Th, T helper; TLR, Toll-like receptor; TNF-α, tumor necrosis factor-alpha; TRPV1, transient receptor potential vanilloid 1; TSLP, thymic stromal lymphopoietin.
